# Relationship of the p22phox (*CYBA*) Gene Polymorphism C242T with Risk of Coronary Artery Disease: A Meta-Analysis

**DOI:** 10.1371/journal.pone.0070885

**Published:** 2013-09-05

**Authors:** Zhijun Wu, Yuqing Lou, Wei Jin, Yan Liu, Lin Lu, Qiujing Chen, Yucai Xie, Guoping Lu

**Affiliations:** 1 Department of Cardiology, Ruijin Hospital, Shanghai Jiao Tong University School of Medicine, Shanghai, China; 2 Department of Pulmonary, Shanghai Chest Hospital, Shanghai Jiao Tong University, Shanghai, China; University Heart Center Freiburg, Germany

## Abstract

**Background:**

Observational and experimental studies have thus far been unable to resolve whether the *CYBA* C242T polymorphism is associated with coronary artery disease (CAD). Therefore, we undertook a comprehensive meta-analysis to more precisely evaluate the influence of this polymorphism on CAD and potential biases.

**Methods:**

We screened MEDLINE, Embase, CNKI, Wanfang and CBM up to January 2013 and extracted data from 22 studies with 9,279 CAD patients and 9,349 controls. A random-effects model was exploited to synthesize the inconsistent outcomes of the individual studies, while addressing between-study heterogeneity and publication bias.

**Results:**

The *CYBA* C242T polymorphism conformed to Hard-Weinberg Equilibrium for all studies (P>0.05). Overall comparison of the T allele with the C allele produced a non-significant risk estimate for CAD but with striking heterogeneity (T versus C: P = 0.87, OR = 0.99, 95%CI 0.89–1.11, P_heterogeneity_<0.0001, I^2^ = 67.8%). However, subgroup analysis by ethnicity documented that the T allele carriers had a marginal risk increase (21%) of CAD among Caucasians (recessive genetic model: P = 0.05, 95%CI 1.00–1.46, P_heterogeneity_ = 0.15, I^2^ = 29.1%). Then data were divided into study design, the significance of CAD risk increase was substantially strengthened in matched case-control studies (allele comparison: P = 0.02, OR = 1.13, 95%CI 1.02–1.26, P_heterogeneity_ = 0.24, I^2^ = 21.6%).Further meta-regression analysis identified that a large proportion of heterogeneity was explained by body mass index (BMI) (P = 0.03, OR = 1.07, 95%CI 1.01–1.15) and study design (P = 0.03, OR = 1.30, 95%CI 1.02–1.64).There was no obvious publication bias as verified by funnel plot and Egger's linear regression test (t = −0.25, P = 0.81 for allele comparison).

**Conclusion:**

Taken together, our results suggested the *CYBA* C242T polymorphism might be a risk-conferring factor on developing CAD and BMI and study design were probable sources of between-study heterogeneity.

## Introduction

Coronary artery disease (CAD) and myocardial infarction (MI) are principal public health contributors to worldwide death and disability, and as such are primary causes of economic burden [1]. About 1 of every 6 deaths was attributed to CAD in the United States in 2008 and as rough estimate 785,000 Americans will have a new coronary attack and 470,000 will have a recurrent attack annually [2]. The multiple potential pathologic pathways of CAD have been widely investigated, ranging from cholesterol accumulation to plaque rupture with thrombus formation [3]. Among these pathways, oxidative stress is thought to play a pivotal role in the pathophysiology of atherosclerosis, which is manifested as increased availability of reactive oxygen species (ROS) and reactive nitrogen species because of an imbalanced redox state. ROS has been implicated in a variety of biological functions, including induction of gene expression and promotion of endothelial cell and smooth muscle cell (SMC) proliferation. Imbalance of ROS production is involved in oxidation of low density lipoprotein, which consequently promotes the progression of atherosclerosis [4], [5].

NAD(P)H oxidase is the predominant cellular source of ROS in the context of atherosclerosis [6]. The phagocyte-specific cytochrome b, as an essential component of NAD(P)H oxidase, plays an important role in regulating NAD(P)H activity and ROS production. Phagocyte-specific cytochrome b is a membrane-associated heterodimer, consisting of 2 subunits (gp91phox and p22phox [CYBA]) [7]. P22phox is essential for vascular cell production of superoxide anion. Human p22phox is encoded by the *CYBA* gene which is located on 16q24. One of the most common polymorphisms of *CYBA* gene is C242T(rs4673), resulting in a non-conservative substitution of histidine for tyrosine at codon 72 and an alteration of NAD(P)H activity by disrupting the heme binding site [8].The C242T polymorphism has been demonstrated to be related to multiple inflammatory diseases [7] and metabolic disorders [9], [10]. Recent epidemiological studies suggested that this polymorphism could be associated with CAD [11], [12], but this could not be confirmed by other studies [13], [14]. The current discordance in reported studies may result from real racial diversity, restrictive sample size and unadjusted environmental confounders. Large meta-analysis makes it possible to achieve a more precise evaluation of this issue. Although a previous meta-analysis of the C242T polymorphism indicated a significant protective effect among Asians, they had a relative small sample size and merely concerned the ethnic difference [15]. In addition, some studies they included were departure from Hardy-Weinberg equilibrium (HWE) [8], [16]. None of these studies had comprehensively compared the effect of the C242T polymorphism on CAD risk in view of study differences. To maximize statistical power and robustly assess the relationship, we have incorporated the published studies to date and meta-analyzed the effect of the C242T polymorphism on CAD risk, while addressing heterogeneity and publication bias. We aimed to systematically estimate the CAD risk of the C242T polymorphism taking different study characteristics into account and go deeply into the origin of the heterogeneity.

## Materials and Methods

### Search strategy

We collected data by searching from PubMed/MEDLINE, Embase, CNKI (China Nation Knowledge Infrastructure Platform), Wanfang and CBM (China Biological Medicine Database) electronic databases up to January 2013. The search strategy followed the guidelines of the 2009 preferred reporting items for systematic reviews and meta-analyses (PRISMA) statement (Checklist S1) [17]. A combination of the following key words was used in the computer-based searches: ‘C242T’ or ‘rs4673’, ‘nicotinamide adenine dinucleotide phosphate oxidase’ or ‘NAD (P) H oxidas’, ‘cytochrome b’ or ‘CYBA’, ‘ph22phox’ and ‘coronary’ or ‘ischemic heart disease’ or ‘myocardial infarction’ or ‘atherosclerosis ’. We scrutinized the full texts of potentially appropriate articles and identified those containing useful data on the topic of interest for inclusion in the meta-analysis. Additional studies were identified by hand-searching and employing the Pubmed related search function. The bibliographies of relevant studies, reviews and previous meta-analyses were screened for additional useful studies which were on the same topic but not found by the initial search. If the allele or genotype frequency was not adequately reported, the original authors were contacted to request the raw data.

### Selection criteria

All studies related to the association of the C242T polymorphism with CAD risk were potentially involved. The following criteria were adopted to the selection: (1) Articles published in English or Chinese journals or their supplements; (2) Studies providing adequate information on the C242T polymorphism by case-control status (studies without controls were excluded); (3) Published articles of human genetics (full texts or abstracts) without ethnicity restriction; (4) If articles involved more than one geographic or racial group, each group was considered as a separate study; (5) If multiple studies stemmed from the same population, only the largest scale study was included to avoid overlapping data; (6) The genotype frequency amongst control must conform to HWE.

Standard definitions were used to diagnosis disease outcomes [18].CAD was defined as previous MI, angina pectoris, or the presence of at least one or more major coronary epicardial coronary vessel with ≥50% luminal obstruction [19]. Acute coronary syndrome (ACS) was defined by unstable angina pectoris and fatal or non-fatal MI [20] and MI defined by a positive cardiac enzymes test in the setting of clinical symptoms and typical electrocardiographic changes [21].

### Data Extraction

We undertook a standard data-collection protocol following the inclusion criteria described above. Two authors (Z.W. and Y. Lou.) independently extracted the relevant characteristics from each selected study and entered the data into separate databases in duplicate. Moreover, the results of data extraction were compared and any encountered divergences were resolved at a consensus meeting. The following information was gathered on the C242T polymorphism on the basis of different cohort: First author's name, publication year, ethnicity, country or area, study design, population source, disease outcomes, matching variables, clinical characteristics for case-patients and controls (such as age, body mass index [BMI], circulating lipid profiles and fibrinogen levels, the proportion of male, hypertension [HTN] , diabetes mellitus [DM] , smoking status and alcohol intake), the distribution of the C242T genotype both in patients and controls and conformity to genotype frequencies with HWE. Continuous variables manifested themselves as mean ± standard deviation (SD) or median (5^th^ and 95^th^ percentiles).

### Quality Assessment

The Newcastle-Ottawa Scale (NOS) [22] was used to assess the methodological quality of all eligible studies. The NOS provides an easy and convenient tool for quality assessment of non-randomized studies based on three broad perspectives: Selection, Comparability, and Exposure for case-control studies. In our meta-analysis, age, gender, BMI and smoking were considered as important confounder factors. A study can be awarded a maximum of one star for each numbered item with the Selection and Exposure categories, and a maximum of two stars for Comparability. Studies can be awarded a maximum score of 9 stars with scores of 5 stars or more regarded to be of medium to high study quality. Quality assessment of each individual study was performed by three authors separately (Z.W., Y. Lou. and Q.C.). Authors W.J. and Y. Liu. were consulted for any encountered disagreement.

### Statistical analysis

The synthetic studies' odd ratios (ORs) corresponding to 95% confidence intervals (CIs) for the relationship between the C242T polymorphism and CAD risk were calculated under different inheritance models, containing allele comparison (T versus C), dominant genetic model (CT+TT versus CC), recessive genetic model (TT versus CT+CC) and homozygote comparison (TT versus CC). We appraised the individual effect size together and modulated the study weights based on in-study variance by exploiting the DerSimonian & Laired method in a random-effects model. The possibility of heterogeneity was also assessed by using the Mantel-Haenszel model [23]. The between-study heterogeneity was estimated using Cochran's chi-square based Q statistic test [24] and P<0.1 was considered significantly heterogeneous among studies. The degree of consistency was estimated via index I^2^ statistic, which determines the percentage of the between-study variability caused by heterogeneity rather than chance. Large values of I^2^ strongly implied the presence of between-study heterogeneity [25], [26].The significance of the pooled OR was evaluated by the Z test, which was considered to be significant for P<0.05. Additionally, we carried out a meta-regression with restricted maximum likelihood estimation to investigate the extent to which covariates explained genetic heterogeneity among the individual ORs. To further assess the origins of inter-study heterogeneity, we examined pre-specified groupings of study characteristics with homogeneous effects, such as ethnicity (Caucasian, Asian and others), study design (matched and not mentioned), population source (hospital-based [H-B] and population-based [P-B]) and endpoints (CAD, ACS and MI).

We undertook a sensitivity analysis by successive one-study removal approach to identify the most influential studies, likely introducing bias to the overall estimation [27]. The visual funnel plot and Egger's test were used to appraise the probability of publication bias. The standard error of log (OR) of CAD risk under the allele comparison was plotted against its OR for each study. The publication bias could be visual by an asymmetric plot and be confirmed by Egger's linear regression test [28]. Egger's test was considered significant for P<0.05. A cumulative meta-analysis might indicate whether initial published research had an impact on subsequent publications and the evolution of the pooled estimates with ascending date of publication.

Consistency of the C242T polymorphism to HWE was examined via the chi-square test or Fisher's exact test based on a Web program (http://ihg2.helmholtz-muenchen.de/cgi-bin/hw/hwa1.pl). Data processing and statistical analyses were performed using Review Manager software release 5.0 (Oxford, England) and Stata 11.0 (Stata Corporation, College Station, Texas, USA). All P values were 2-sided.

## Results

### Flow of included studies

**Figure 1:**
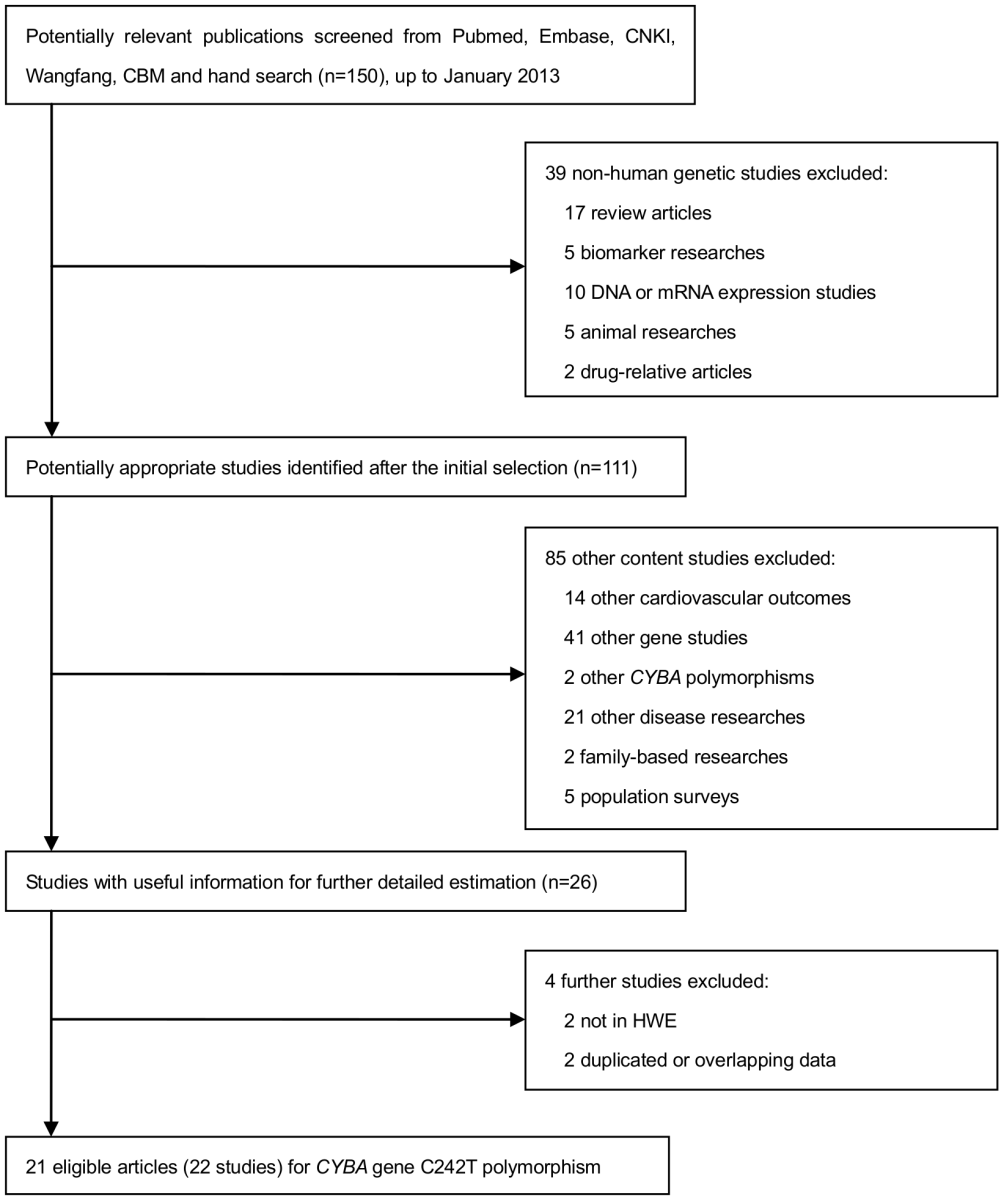
Flow diagram of search strategy and study selection for meta-analysis.

The flow diagram schematizing the selection process of included studies is shown in Figure 1. A total of 150 relevant publications were retrieved in accordance with our search strategy and inclusion criteria. After applying selection criteria, 124 studies were removed. The data from 26 studies depicting the association between the C242T polymorphism and CAD risk were pooled into the provisional data set. Among these studies, 2 data sets conducted in the Chinese [29] and Japanese population [10] were overlapped by other studies with larger cohort [11], [30]. Moreover, the genotyping frequency in the control population of Mata-Balaguer *et al.* [16] and Inoue *et al.* [8] did not conform to HWE (P_HWE_ = 0.034 & 0.029); these two studies were precluded. Because the study by Saha *et al.* [31] contained data from two different racial descents (Chinese & Indian), we treated them as two independent studies when estimating the individual effect sizes. The Indian population was classified into the “others” group as its lineage was miscellaneous and cannot simply be grouped as Asian or Caucasian [32]–[34].Because the study population used in Li *et al.* [14] was of mixed background (82.9% of Caucasian), this study together with 2 Indian studies [31], [35], were collapsed into the “others” group.

In total, 21 publications involving 22 studies (20 articles written in English [11], [13], [14], [30], [31], [35]–[49] and 1 in Chinese) with sufficient information fulfilled our search strategy. Of the 22 qualifying studies, 13 used a Caucasian population [13], [36]–[39], [41]–[48], 6 used an Asian population [11], [30], [31], [40], [49] and 3 performed in others [14], [31], [35]. All studies were published between 1999 and 2012 and utilized a retrospective case-control design .P-B controls were adopted in 6 studies [31], [37], [41], [45], [49] and H-B in 16 studies [11], [13], [14], [30], [35], [36], [38]–[40], [42]–[44], [46]–[48]. CAD patients and controls were matched for age and gender in 11 studies [13], [31], [39], [41]–[43], [45]–[47], while the other 11 failed to mention this in their study design [11], [14], [30], [35]–[38], [40], [44], [48], [49]. CAD was employed as the primary outcome in 16 of the studies [13], [14], [30], [31], [35]–[38], [43]–[46], [48], [49], while MI and ACS were taken as endpoints in 4 [11], [39], [40], [47] and 2 studies [41], [42], respectively.

### Quantitative data synthesis

Our meta-analysis encompassed 22 studies with 9,279 CAD patients and 9,349 controls. The selected characteristics of these eligible studies were shown in Table 1. The allele and genotype frequencies of the C242T polymorphism of each individual study which corresponded to HWE(P_HWE_>0.05) are also listed in Table 2. The combined overall frequency of the T allele was 23.8% in cases and 18.0% in controls. The frequency of the T allele among Caucasians (34.3% cases, 33.5% controls) was markedly higher than that among Asians (8.7% cases, 9.5% controls). The T allele proportion in the “other” group (35.9% cases, 64.1% controls) was similar to that in the Caucasian group. All the 22 studies were assessed according to the NOS scale and the results of the quality assessment are summarized in Table 3. Most studies (82%) scored 5 stars or more, suggesting a moderate to good quality.

**Table 1:** The baseline characteristics of all qualified studies included in the meta-analysis.

First Author	Year	Ethnicity	geographic location	Source	Endpoint	Study design	Status	Age, year	Gender, M(%)	HTN,%	DM,%	Smoke,%	BMI, kg/m^2^
Cai H	1999	Caucasian	Australia	H-B	CAD	not mentioned	cases	56.9±7.0	80	46.3	13.6	71.2	28.2±4.7
							controls	54.5±8.3	46.8	35.8	5.9	52.6	27.6±4.7
Fan M	2006	Caucasian	Finland	P-B	CAD	not mentioned	cases	62±10	77	95	29	59	27.3±4.1
							controls	55±11	58	95	16	52	27.2±4.6
Fang SX	2012	Asian	China	H-B	CAD	matched	cases	50.6±7.4^a^	68.6	46.5	–c	39.5	–
								68.5±7.7^b^	–	–	–	–	–
							controls	49.6±8.7	73	36.2	–	46.7	–
Gardemann A	1999	Caucasian	Germany	H-B	CAD	not mentioned	cases	62.7±9.3	–	65	20	–	26.9±3.3
							controls	58.5±10.5	–	54	11	–	26.9±3.5
Goliasch G	2011	Caucasian	Vienna	H-B	MI	matched	cases	37.3(33.8–39)	87.1	42	30	78	27.5(24.3–29.8)
							controls	34.7(30.6–38.3)	90	17.5	12.5	43.1	24.6(22.4–27.8)
He MA	2007	Asian	China	H-B	CAD	not mentioned	cases	64.7±10.3	57	64.6	21.59	49.2	24.2±3.5
							controls	61.9±7.3	51.6	34.6	6.57	37.4	24.6±3.4
Katakami N	2010	Asian	Japan	H-B	MI	not mentioned	cases	62.2±9.4	64.2	82.3	100	45.1	24.0±3.5
							controls	59.5±10.5	60.6	72.9	100	43.2	24.2±3.8
Lee WH	2001	Asian	Korea	P-B	CAD	not mentioned	cases	56.1±10.7	100	37.1	19	79	24.4±3.2
							controls	50.2±10.1	100	7	5.1	75.8	23.9±2.7
Li A	1999	Other	US	H-B	CAD	not mentioned	cases	60±0.8	76.5	47.7	27.5	12.1	–
							controls	57±1.1	47.6	40.8	8.7	14.6	–
Macias-Reyes A	2008	Caucasian	Spain	P-B	ACS	matched	cases	56±10	78	–	33.9	50	27.2±3.7
							controls	54.5±11	73.7	–	12.1	27.3	27.3±3.8
Morgan TM	2007	Caucasian	US	H-B	ACS	matched	cases	60.7±12.5(M)	67.8	60.1	23.8	33.1	29.1±5.5(M)
								63.1±13.2(F)	–	–	–	–	29.9±6.9(F)
							controls	60.0±12.1(M)	60.6	51.2	11.9	13.2	27.9±5.0(M)
								61.8±12.8(F)	–	–	–	–	27.7±6.9(F)
Najafi M	2012	Caucasian	Iran	H-B	CAD	matched	cases	62.8±11.9	67.5	–	–	24.6	25.5±4.3
							controls	55.9±13.9	32.4	–	–	17.7	26.2±6.2
Narne P	2012	Others	India	H-B	CAD	not mentioned	cases	–	–	–	100	–	–
							controls	–	–	–	100	–	–
Nasti S	2006	Caucasian	Italia	H-B	CAD	not mentioned	cases	65.5±10.0	83.2	69.1	26.2	71.6	26.2±3.3
							controls	62.2±14.8	67.9	56.3	13.5	77.2	26.0±4.4
Niemiec P	2007	Caucasian	Poland	P-B	CAD	matched	cases	43.8±5.9	66.9	59.3	5.8	57.6	27.0±4.3
							controls	34.2±10.4	69.2	4.1	0	32	24.6±3.9
Nikitin AG	2009	Caucasian	Russia	H-B	CAD	matched	cases	59.2±7.9	54.3	29.3	0	21.6	28.4±5.1
							controls	55.5±9.0	56.8	41.7	0	11.4	29.2±6.4
Saha N[Chinese]	1999	Asian	Singapore	P-B	CAD	matched	cases	55.3±9.8	100	–	28.5	61.6	23.9±3.7
							controls	54.5±11.6	100	–	0	33.6	23.9±3.9
Saha N[Indian]	1999	Others	Singapore	P-B	CAD	matched	cases	55.2±7.9	88.1	–	44.4	43.7	24.9±3.3
							controls	53.9±12.4	92.9	–	0	13	24.3±3.7
Stanger O	2001	Caucasian	Austria	H-B	CAD	matched	cases	52.9±5.8	100	40.7	0	60.2	26.6±2.9
							controls	54.2±7.4	100	33.3	0	22.2	25.5±3.3
Vasiliadou C	2007	Caucasian	Greece	H-B	MI	matched	cases	46.9±1.1	–	–	–	–	–
							controls	–	–	–	–	–	–
Yamada Y	2002	Asian	Japan	H-B	MI	not mentioned	cases	62.1±10.1	100	47	34.7	57.8	23.6±2.9
							controls	62.0±10.4	100	57.3	16.2	55.2	23.6±2.7
Zafari AM	2002	Caucasian	US	H-B	CAD	not mentioned	cases	56.4±17.4^d^	98.8	70.1	39	79.9	–
								63.2±9.9^e^	–	–	–	–	–
							controls	57.1±9.3	94.2	63.5	28.8	63.5	–

P-B: population-based study; H-B: hospital-based study; CAD: coronary artery disease; MI: myocardial infarction; ACS: acute coronary syndrome; M(%): male(percent); F: female; HTN: hypertension; DM: diabetes mellitus; BMI: body mass index;

a :early-onset coronary artery disease;

b :late-onset coronary artery disease;

c :data not available;

d :single-vessel disease;

e :multi-vessel disease; Age and BMI are expressed as mean ±SD (standard deviation) or median (5th and 95th percentiles).

**Table 2:** Sample size, the distribution of the C242T allele frequencies and genotypes among CAD patients and controls,and P value of HWE in controls.

First Author	Sample size		T allele,%		C allele,%		TT genotype		CT genotype		CC genotype		HWE,
	cases	controls	cases	controls	cases	controls	cases	controls	cases	controls	cases	controls	P value
Cai H	550	139	34.5	31.7	65.5	68.3	65	13	250	62	235	64	0.72
Fan M	250	152	18.0	25.0	82.0	75.0	12	10	66	56	172	86	0.83
Fang SX	746	470	9.5	6.5	90.5	93.5	4	0	134	61	608	409	0.13
Gardemann A	1706	499	34.1	33.9	65.9	66.1	207	65	748	208	751	226	0.12
Goliasch G	102	200	34.8	36.7	65.2	63.3	11	31	47	79	41	82	0.11
He MA	565	609	3.2	5.6	96.8	94.4	2	2	32	64	531	543	0.94
Katakami N	226	3593	8.0	9.5	92.0	90.5	0	33	36	618	190	2942	0.93
Lee WH	305	215	7.9	11.9	92.1	88.1	4	1	40	49	261	165	0.19
Li A	149	103	41.6	34.0	58.4	66.0	27	14	70	42	52	47	0.36
Macias-Reyes A	304	315	38.5	37.8	61.5	62.2	48	47	137	145	119	123	0.69
Morgan TM	811	650	34.7	33.7	65.3	66.3	121	74	271	293	347	288	0.97
Najafi M	114	68	40.8	41.2	59.2	58.8	23	12	47	32	44	24	0.81
Narne P	160	121	27.5	40.5	72.5	59.5	13	22	62	54	85	45	0.42
Nasti S	276	218	40.0	33.3	60.0	66.7	37	25	147	95	92	98	0.79
Niemiec P	172	169	37.8	35.8	62.2	64.2	25	18	80	85	67	66	0.22
Nikitin AG	313	132	25.4	18.6	74.6	81.4	34	4	91	41	188	87	0.75
Saha N[Chinese]	151	167	9.6	9.3	90.4	90.7	3	3	23	25	125	139	0.15
Saha N[Indian]	126	154	39.7	38.0	60.3	62.0	17	20	66	77	43	57	0.45
Stanger O	108	45	37.0	40.0	63.0	60.0	15	6	50	24	43	15	0.46
Vasiliadou C	197	204	42.1	33.6	57.9	66.4	34	21	98	95	65	88	0.53
Yamada Y	1784	1074	10.3	12.8	89.7	87.2	25	16	319	242	1440	815	0.68
Zafari AM	164	52	37.6	38.0	62.4	62.0	21	6	70	26	58	18	0.46
Total	9279	9349	23.8	18.0	76.2	82.0	748	443	2884	2473	5557	6427	

HWE: Hardy–Weinberg equilibrium. The P-value of HWE determined by the χ^2^ test or Fisher's exact test among controls.

**Table 3:** Quality assessment for all the included studies according to the Newcastle-Ottawa Scale.

		Quality Indicators		
First Author	Year	Selection	Comparability	Exposure
Cai H	1999	☆☆☆	☆	☆☆
Fan M	2006	☆☆☆☆	☆	☆☆
Fang SX	2012	☆☆	☆☆	☆
Gardemann A	1999	☆☆	☆	☆☆
Goliasch G	2011	☆☆☆	☆☆	☆
He MA	2007	☆☆☆	☆	☆
Katakami N	2010	☆☆	☆	☆
Lee WH	2001	☆☆☆	☆	☆
Li A	1999	☆☆	☆	☆
Macias-Reyes A	2008	☆☆☆☆	☆☆	☆☆
Morgan TM	2007	☆☆☆	☆☆	☆
Najafi M	2012	☆☆☆	☆☆	☆
Narne P	2012	☆☆☆		☆☆
Nasti S	2006	☆☆☆	☆	☆☆
Niemiec P	2007	☆☆☆☆	☆☆	☆
Nikitin AG	2009	☆☆☆	☆☆	☆
Saha N[Chinese]	1999	☆☆☆☆	☆☆	☆
Saha N[Indian]	1999	☆☆☆☆	☆☆	☆
Stanger O	2001	☆☆	☆☆	☆
Vasiliadou C	2007		☆☆	
Yamada Y	2002	☆☆☆	☆	☆
Zafari AM	2002	☆☆☆		☆

**Figure 2:**
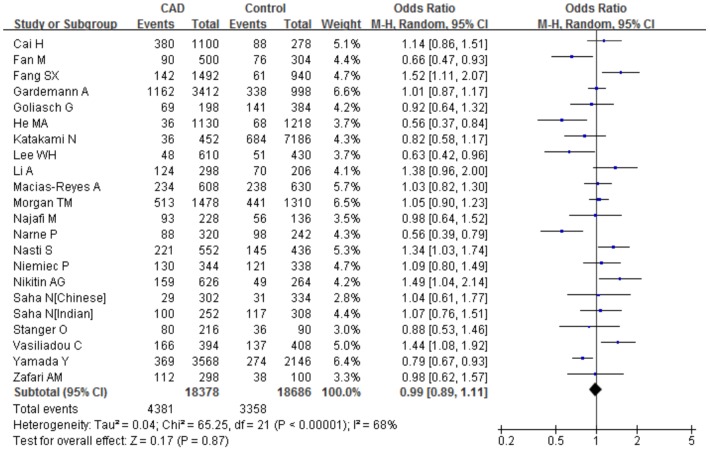
Meta-analysis for the overall association between the *CYBA* C242T polymorphism and CAD under the allele comparison (T versus C). ‘Events’ indicates the total number of T allele. ‘Total’ indicates the total number of T allele plus C allele.

Furthermore, we assessed the association of the C242T polymorphism with CAD risk under different genetic models. The summary results of the meta-analysis were shown in Table 4. There was no predominant association between the C242T polymorphism and CAD produced by comparing the T allele with C allele overall (allele comparison: P = 0.87, OR = 0.99, 95%CI 0.89–1.11; dominant model: P = 0.58, OR = 0.96, 95%CI 0.84–1.10; recessive model: P = 0.15, OR = 1.14, 95%CI 0.95–1.36; homozygote comparison: P = 0.20, OR = 1.14, 95%CI 0.93–1.40) (Figure 2). We identified a large between-study heterogeneity across all genotypic models (allele comparison: I^2^ = 67.8%, P_heterogeneity_<0.0001; dominant model: I^2^ = 65.7%, P_heterogeneity_<0.0001; homozygote comparison: I^2^ = 35.7%, P_heterogeneity_ = 0.05), with the exception of the recessive model (I^2^ = 27.9%, P_heterogeneity_ = 0.11) . Because every subject contributes two observations under the allele comparison and we cannot a priori hypothesis that these two observations are independent, we computed log ORs with standard errors in each study and then used them in our meta-analysis. As a result, the risk estimates did not change if the clustering of the alleles in each subject was taken into account.

**Table 4:** Summary estimates for ORs and 95% CI in different subgroups under various genetic contrasts.

Genotype contrasts	study population	study number, (case/control),n(n/n)	P_heterogeneity_	I^2^,%	P value^a^	OR	95% CI
*Total studies*							
Allele comparison		22(9279/9349)	0.00	67.8	0.87	0.99	0.89–1.11
(T versus C)							
Dominant model		22(9279/9349)	0.00	65.7	0.58	0.96	0.84–1.10
(CT+TT versus CC)							
Recessive model		22(9279/9349)	0.11	27.9	0.15	1.14	0.95–1.36
(TT versus CT+CC)							
Homozygote comparison		22(9279/9349)	0.05	35.7	0.20	1.14	0.93–1.40
(TT versus CC)							
*Ethnicity*							
Allele comparison	Caucasian	13(5067/2843)	0.06	41.0	0.19	1.07	0.97–1.19
	Asian	6(3777/6128)	0.00	75.6	0.24	0.85	0.64–1.12
	Others	3(435/378)	0.00	84.8	0.81	0.94	0.56–1.59
Dominant model	Caucasian	13(5067/2843)	0.05	43.5	0.58	1.04	0.90–1.20
	Asian	6(3777/6128)	0.00	77.4	0.21	0.82	0.61–1.12
	Others	3(435/378)	0.01	80.1	0.93	0.97	0.51–1.84
Recessive model	Caucasian	13(5067/2843)	0.15	29.1	0.05	1.21	1.00–1.46
	Asian	6(3777/6128)	0.65	0.0	0.89	1.04	0.61–1.76
	Others	3(435/378)	0.04	69.0	0.65	0.84	0.40–1.76
Homozygote comparison	Caucasian	13(5067/2843)	0.16	28.3	0.06	1.22	0.99–1.49
	Asian	6(3777/6128)	0.65	0.0	0.96	0.99	0.58–1.67
	Others	3(435/378)	0.01	80.7	0.76	0.85	0.31–2.33
*Study design*							
Allele comparison	matched	11(3144/2574)	0.24	21.6	0.02	1.13	1.02–1.26
	not mentioned	11(6135/6775)	0.00	75.0	0.11	0.87	0.73–1.03
Dominant model	matched	11(3144/2574)	0.23	22.9	0.22	1.09	0.95–1.26
	not mentioned	11(6135/6775)	0.00	75.5	0.15	0.85	0.69–1.06
Recessive model	matched	11(3144/2574)	0.26	19.1	0.02	1.32	1.06–1.66
	not mentioned	11(6135/6775)	0.37	8.1	0.75	0.97	0.78–1.20
Homozygote comparison	matched	11(3144/2574)	0.29	16.0	0.03	1.31	1.03–1.66
	not mentioned	11(6135/6775)	0.07	41.8	0.89	0.98	0.71–1.34
*Population source*							
Allele comparison	P-B	6(1308/1172)	0.08	48.4	0.35	0.91	0.75–1.11
	H-B	16(7971/8177)	0.00	72.4	0.77	1.02	0.89–1.17
Dominant model	P-B	6(1308/1172)	0.09	47.7	0.19	0.85	0.66–1.08
	H-B	16(7971/8177)	0.00	69.8	0.95	1.00	0.85–1.19
Recessive model	P-B	6(1308/1172)	0.80	0.0	0.53	1.10	0.82–1.47
	H-B	16(7971/8177)	0.03	43.8	0.25	1.15	0.91–1.45
Homozygote comparison	P-B	6(1308/1172)	0.74	0.0	0.68	1.07	0.78–1.46
	H-B	16(7971/8177)	0.01	49.5	0.26	1.16	0.90–1.51
*Endpoint*							
Allele comparison	CAD	16(5855/3313)	0.00	69.5	0.88	0.99	0.85–1.15
	MI	4(2309/5071)	0.01	77.0	0.78	0.96	0.72–1.28
	ACS	2(1115/965)	0.91	0.0	0.53	1.04	0.92–1.19
Dominant model	CAD	16(5855/3313)	0.00	68.5	0.65	0.96	0.79–1.16
	MI	4(2309/5071)	0.02	71.5	0.90	0.98	0.71–1.35
	ACS	2(1115/965)	0.55	0.0	0.34	0.92	0.77–1.10
Recessive model	CAD	16(5855/3313)	0.23	19.6	0.38	1.10	0.89–1.37
	MI	4(2309/5071)	0.10	52.1	0.99	1.00	0.56–1.80
	ACS	2(1115/965)	0.18	43.2	0.11	1.33	0.94–1.88
Homozygote comparison	CAD	16(5855/3313)	0.07	37.4	0.36	1.13	0.87–1.49
	MI	4(2309/5071)	0.06	59.6	0.90	1.05	0.53–2.06
	ACS	2(1115/965)	0.39	0.0	0.10	1.25	0.96–1.64

a :Test for overall effect;

P-B:population-based; H-B: hospital-based; CAD: coronary artery disease; MI: myocardial infarction; ACS: acute coronary syndrome.

### Sensitivity analysis

The striking heterogeneity identified across studies could influence the total outcome of the meta-analysis. Therefore, we performed a sensitivity analysis to look for the study or studies making the greatest contribution to heterogeneity. No single study or group of studies could be confirmed as disproportionately influencing the heterogeneity and ORs in total and subgroup analysis.

### Cumulative analysis and publication bias

**Figure 3:**
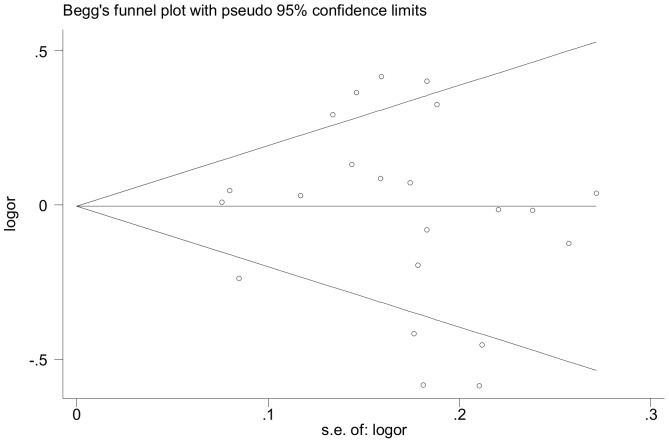
Begg's funnel plot analysis to detect publication bias for allele comparison (T versus C) of the C242T polymorphism.

We did not find any clear evidence to suggest that the first published study affected and brought about the ensuing replication via cumulative meta-analysis (data not shown). In addition, funnel plot analysis suggested that the overall results were not asymmetric and Egger's regression asymmetry test further verified an absence of publication bias (t = −0.25, P = 0.81 for allele comparison) in the overall estimates (Figure 3).

### Subgroup analysis

**Figure 4:**
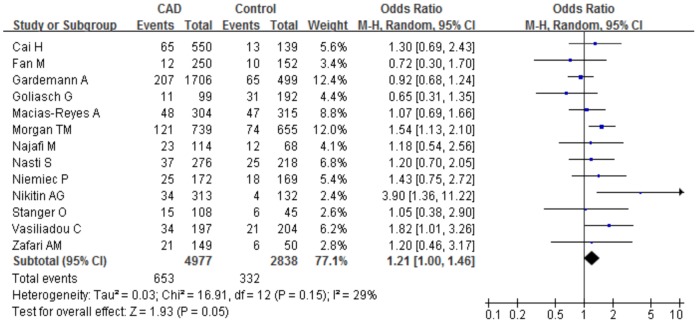
Meta-analysis for the association between the C242T polymorphism and CAD risk among Caucasians. The TT homozygote shows a borderline increased risk of CAD under the recessive model. ‘Events’ indicates the total number of TT genotype. ‘Total’ indicates the total number of TT genotype plus TT+ CT genotype.

In view of a conspicuous heterogeneity present in total analysis, we undertook a panel of subgroup analyses on ethnicity, population source, study design and endpoint type. The initial aim was to evaluate the possible variability of overall estimates due to ethnicity and data was classified in accordance with the 3 racial descent groups: Caucasian (13 studies recruited 5,067 cases and 2,843 controls), Asian (6 studies recruited 3,777 cases and 6,128 controls), and others (3 study recruited 435 cases and 378 controls). The ORs of the C242T polymorphism appeared to decrease in the Asian group (allele comparison: P = 0.24, OR = 0.85, 95%CI 0.64–1.12, P_heterogeneity_ = 0.001, I^2^ = 75.6%; dominant model: P = 0.21, OR = 0.82, 95%CI 0.61–1.12, P_heterogeneity_<0.0001, I^2^ = 77.4%) and in others group (allele comparison: P = 0.81, OR = 0.94, 95%CI 0.56–1.59, P_heterogeneity_ = 0.001, I^2^ = 84.8%; dominant model: P = 0.93, OR = 0.97, 95%CI 0.51–1.84, P_heterogeneity_ = 0.01, I^2^ = 80.1%) without significance. Interestingly, the risk estimates of the C242T polymorphism with CAD differed between Caucasians and Asians, with an increased OR of CAD among the former (allele comparison: P = 0.19, OR = 1.07, 95%CI 0.97–1.19, P_heterogeneity_ = 0.06, I^2^ = 41%; dominant model: P = 0.58, OR = 1.04, 95%CI 0.90–1.20, P_heterogeneity_ = 0.05, I^2^ = 43.5%). In particularly, we observed a marginal increased risk of CAD under the recessive genetic model (P = 0.05, OR = 1.21, 95%CI 1.00–1.46, P_heterogeneity_ = 0.15, I^2^ = 29.1%) and in homozygote comparison (P = 0.06, OR = 1.22, 95%CI 0.99–1.49, P_heterogeneity_ = 0.16, I^2^ = 28.3%) among Caucasians without distinct heterogeneity (Figure 4).

**Figure 5:**
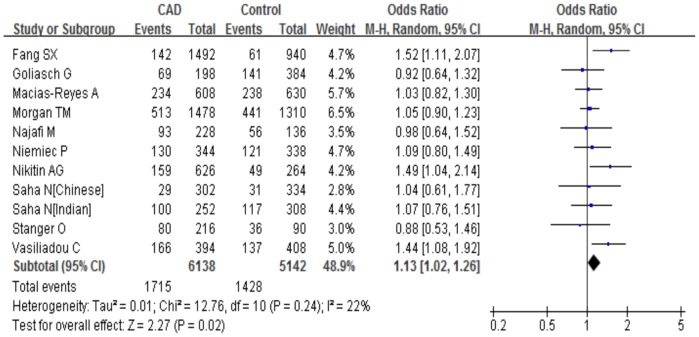
Meta-analysis for the association between the C242T polymorphism and CAD risk in matched studies. The C242T polymorphism shows a significant increased risk of CAD under the allele comparison (T versus C). ‘Events’ indicates the total number of T allele. ‘Total’ indicates the total number of T allele plus C allele.

To address study design, stratification by the matched information for age and gender between cases and controls was performed, as a result, the T allele carriers showed a remarkable increased risk of CAD under most genetic models (allele comparison: P = 0.02, OR = 1.13, 95%CI 1.02–1.26, P_heterogeneity_ = 0.24, I^2^ = 21.6%; recessive model: P = 0.02, OR = 1.32, 95%CI 1.06–1.66, P_heterogeneity_ = 0.26, I^2^ = 19.1%, homozygote comparison: P = 0.03, OR = 1.31, 95%CI 1.03–1.66, P_heterogeneity_ = 0.29, I^2^ = 16%) but not under the dominant model (P = 0.22, OR = 1.09, 95%CI 0.95–1.26, P_heterogeneity_ = 0.23, I^2^ = 22.9%) in matched studies (Figure 5). Although the ORs of the C242T polymorphism seem to be reversed in studies which did not specify whether the matched case-control study design was used (allele comparison: P = 0.11, OR = 0.87, 95%CI 0.73–1.03, P_heterogeneity_<0.0001, I^2^ = 75%; dominant model: P = 0.15, OR = 0.85, 95%CI 0.69–1.06, P_heterogeneity_<0.0001, I^2^ = 75.5%; recessive model: P = 0.75, OR = 0.97, 95%CI 0.78–1.20, P_heterogeneity_ = 0.37, I^2^ = 8.1%, homozygote comparison: P = 0.89, OR = 0.98, 95%CI 0.71–1.34, P_heterogeneity_ = 0.07, I^2^ = 41.8%), this was not statistically significant. In further subgroup analysis, a lack of dramatic association of the C242T polymorphism with CAD risk was returned when using any genetic model in which data were categorized according to population sources and disease outcomes (Table 4).

### Meta-regression analysis

We performed a univariate meta-regression analysis for pre-defined underlying sources of heterogeneity, including ethnicity, population source, case definition, study-design, the baseline characteristics of total population (such as age, gender, BMI, the proportion of HTN, DM and smoking status). The adjusted R^2^ statistic indicates the proportion of between-study variance explained by the covariates. Accordingly, a large proportion of heterogeneity was significantly attributed to BMI (P = 0.03) and study design (P = 0.03) respectively, with CAD risk of the T allele being increasing in high BMI population (OR = 1.07, 95%CI 1.01–1.15) compared to low BMI population and in matched studies (OR = 1.30, 95%CI 1.02–1.64) compared to studies which did not mention whether the matched case-control study design was used (Table 5).

**Table 5:** Results from the meta-regreesion analysing the association of potential factors with the CAD risk.

	OR	95% CI	P_COV_ a	R^2^,%
*Univariate meta-regression*				
Ethnicity	1.215	0.939–1.572	0.130	12.46
Population source	1.126	0.835–1.519	0.416	−2.66
Study design	1.296	1.024–1.640	0.033	25.13
Endpoint	1.001	0.750–1.335	0.996	−8.99
Male, %	0.997	0.989–1.004	0.366	2.24
Age, year	0.999	0.981–1.016	0.861	−10.45
Smoking, %	0.995	0.986–1.002	0.170	5.28
HTN, %	0.996	0.988–1.005	0.401	−1.25
DM,%	0.995	0.991–1.000	0.050	33.83
BMI,kg/m^2^	1.074	1.008–1.145	0.029	50.82
*The pooled effect estimate after adjustment for ethnicity*				
Population source	1.114	0.833–1.490	0.244	9.94
Study design	1.250	1.978–1.597	0.061	27.69
Endpoint	1.018	0.770–1.345	0.327	3.71
Male, %	0.997	0.989–1.005	0.485	−0.41
Age, year	1.000	0.982–1.018	0.677	−11.40
Smoking, %	0.995	0.988–1.002	0.146	6.72
HTN, %	0.996	0.987–1.005	0.396	3.04
DM,%	0.997	0.992–1.002	0.064	45.53
BMI,kg/m^2^	1.030	0.916–1.158	0.067	56.16
*The pooled effect estimate after adjustment for BMI*				
Ethnicity	1.201	0.781–1.847	0.067	56.16
Population source	1.039	0.812–1.329	0.099	39.17
Study design	1.133	0.903–1.422	0.054	38.47
Endpoint	1.056	0.839–1.329	0.091	43.04
Male, %	1.001	0.994–1.009	0.101	37.61
Age, year	0.997	0.982–1.012	0.094	40.22
Smoking, %	0.999	0.991–1.001	0.120	26.94
HTN, %	0.997	0.989–1.004	0.065	46.93
DM,%	0.999	0.993–1.005	0.114	40.79

HTN:hypertension; DM:diabetes mellitus; BMI:body mass index; Age and BMI were considered as continuous variables.

a :P values for significance of covariates in the pooled genetic effect.

Furthermore, we compared the descriptive factors between Caucasian and non-Caucasian studies to investigate underlying confounders which may influence the results (Table 6).We found the distribution in study design and BMI was distinctly different between the two groups and could be a likely source of potential confounders. The proportion of Caucasian studies using matched study design (61.5%) was almost double that of non-Caucasian studies (33.3%). The out point of the BMI (25.8 kg/m^2^) was chosen as the median. Interestingly, most Caucasian studies (81.8%) had BMI≥25.8 kg/m^2^, while none of non-Caucasian studies had BMI≥25.8 kg/m^2^.

**Table 6:** The percentage distribution of descriptive factors in Caucasian studies and non-Caucasian studies.

	Ethnicity,%	
Descriptive factors	Caucasian	non-Caucasian
matched study design	61.5	33.3
Hospital-based controls	76.9	66.7
CAD outcome	69.2	77.8
BMI≥25.8 kg/m^2^	81.8	0

We took ethnicity and BMI into account by separately adding them into the multivariable regression. There was a decrease in the OR increase of population source, study design and BMI and a slight increase in OR increase of disease endpoint after adjustment for Caucasian/non-Caucasian, which was similar to that after adjustment for BMI. There were no substantial changes observed in the effect estimates of age, gender, HTN, DM and smoking status after adjusting for ethnicity and BMI. Nevertheless, the adjusted effects did not attain significance. The frequent reduction by adjusting may imply that confounding of the potential factors by ethnicity and BMI was likely to overestimate the influence of the study factors on the CAD risk estimates [50]. We also found that the adjusted R^2^ value significantly increased after adjustment for BMI.

## Discussion

The correlation of the C242T polymorphism with CAD risk has yet to be clarified. Reckoning the CAD risk of the minor allele in independent case-control studies generated a conspicuous divergence, which was attributed to such causes as racial diversity, research strategy and limited sample size [51]. To address these issues, we commenced a meta-analysis and provided a comprehensive assessment of the publically available studies [52]. The current meta-analysis, including a total of 9,279 cases and 9,349 controls, is one of the largest systematic reviews addressing the association between the C242T polymorphism and CAD risk. Consequently, the overall comparison of T allele with C allele yielded a non-significant risk for CAD under different genetic models incorporating distinguishable heterogeneity.

Given that distinct heterogeneity was present and could not be completely eliminated [53], we conducted a subgroup analysis to investigate the potential effect of the C242T polymorphism on different groups with homogenous characteristics. As a result, we verified that ethnicity is an underlying source of between-study heterogeneity. In fact, the risk estimates of the C242T polymorphism on CAD among Caucasians were dramatically different to that among Asians. We observed a borderline increased risk of CAD under the recessive model and the homozygote comparison in the Caucasian group, while the opposite trend was observed among Asians. This discrepancy could be attributed to the high variation in C242T allele frequency across different ethnic populations. The T allele frequency among Caucasians was approximately fourfold higher than that among Asians. In addition, we speculate that the pleiotropic effect of the C242T polymorphism due to various genetic ancestral backgrounds could be another explanation. Our finding are inconsistent with a previous meta-analysis involving 6,273 cases and 5,045 controls [15]. The previous meta-analysis by Fang *et al.* [15] showed that the T allele carriers had a significant protective effect among Asians but not among Caucasians. This discrepancy is probably due to their relative small sample size and inadequate statistical power. Another explanation could be that the Fang *et al.* [15] meta-analysis included the studies by Mata-Balaguer *et al.* [16] and Inoue *et al.* [8], which significantly violated HWE. Deviations of HWE in control group are likely owing to genotyping errors, population classification and selection bias in the choice of control population and environmental exposures, resulting in reduced precision and raising doubt about the validity in estimation of effect sizes [54]. Hence, we excluded these two studies in order to avoid biasing the overall estimates in our meta-analysis. It is essential to set up an optimized databank of the C242T polymorphism concerning CAD in defined racial groupings to ascertain the reliability of our results.

As well as the obvious association to ethnicity, other environment exposures have confounding potential for the interpretation of between-study heterogeneity. When taking multiple study-level covariates from clinical characteristics into account, we identified study design and BMI as exhibiting significance and positivity in univariate meta-regression analysis, and as such should be cautiously deciphered. Moreover, we found that there was a larger proportion of the Caucasian studies using matched study design compared to non-Caucasian studies. The studies among Caucasians were also more often performed in high BMI populations than those among non-Caucasians. Then accounting for the effects of ethnicity and BMI in the multivariable meta-regression, study design was no longer detected as a significant source of heterogeneity. Therefore, study design and BMI are likely confounders. The estimated advantage of Caucasians over non-Caucasians may be partially accounted for by the effect of study design and BMI and therefore, the effect of ethnicity on the variation cannot be regarded as an independent factor. Although meta-regression suggests an ecological correlation rather than a causal inference, our result implies underlying confounding of the environmental factors with genotype. Large amounts of observational studies have verified that increased BMI contributes causally to CAD [55]–[57]. Considering a relative wide range of confidence intervals in our results, further carefully designed genetic association studies involving the interaction of BMI are needed to more accurately estimate the risk evaluation of CAD.

Although the relatively large sample size and conformation to HWE strengthen our meta-analysis [58], there are some methodology limitations [59]. First, large positive results are more likely to be accepted for publication than small negative results. Therefore, it was likely that the unpublished studies and “grey” literature (articles in languages other than English and Chinese) were not included. Although the Egger's test and funnel plots revealed that an absence of publication bias in our meta-analysis, this could not be ruled out completely. Second, some H-B studies recruited controls from an ill-defined background, which could not represent the true exposure experience of the source population and probably resulted in a biased estimate of the ORs. Third, our meta-analysis was specific to a single polymorphism on the *CYBA* gene and did not account for possible synergistic effects of other candidate genes or polymorphisms. Genetic susceptibility to CAD is most likely based on the interaction of polygenic and environmental factors [60], [61] and optimized models concerning these aspects are required.

Experimental studies have suggested the C242T polymorphism affects the heme-binding site and alters electron flow and superoxide production [62], which results in decreased basal and NAD(P)H-stimulated ROS production. Therefore, reduced ROS levels in the atherosclerotic vessels might be a sensitive signal of declined SMC survival. SMC-depleted plaques tend to be more vulnerable which is characterized by erosion or disruption of their caps [63], [64].Our results complement the previous evidences suggesting that a decrease in NAD(P)H activity, and thus decreased ROS production, could also accelerate the progression of atherosclerosis. Further confirmation of our results is needed before it is possibly to link p22phox to the development of atherosclerosis.

Taken together, our meta-analysis, comprising 18,628 participants, implied that the T allele might carry an increased risk of CAD. However, it is conceivable that the C242T polymorphism has a heterogeneous effect on CAD across different ethnicities, being moderate among Caucasians but lacking significance among Asians. Our meta-analysis also highlights that it is necessary to consider potential gene-gene and gene-environment interactions when trying to interpret and combine observed data. For now,it remains unclear whether the T allele carriers have positive or negative effects on CAD and further population surveys and functional researches will be required to elucidate the true relationship.

## Supporting Information

Checklist S1 **The PRISMA checklist for this meta-analysis.**
(DOC)
