# Correction: Relationship of the p22phox (*CYBA*) Gene Polymorphism C242T with Risk of Coronary Artery Disease: A Meta-Analysis

**DOI:** 10.1371/annotation/7f489b96-e43f-4c6d-86d7-0689c8a55eba

**Published:** 2013-12-11

**Authors:** Zhijun Wu, Yuqing Lou, Wei Jin, Yan Liu, Lin Lu, Qiujing Chen, Yucai Xie, Guoping Lu

A grant from the National Natural Science Foundation of China was incorrectly omitted from the Funding Statement. The number of the grant is: 81170122. 

